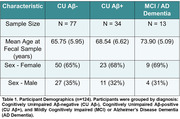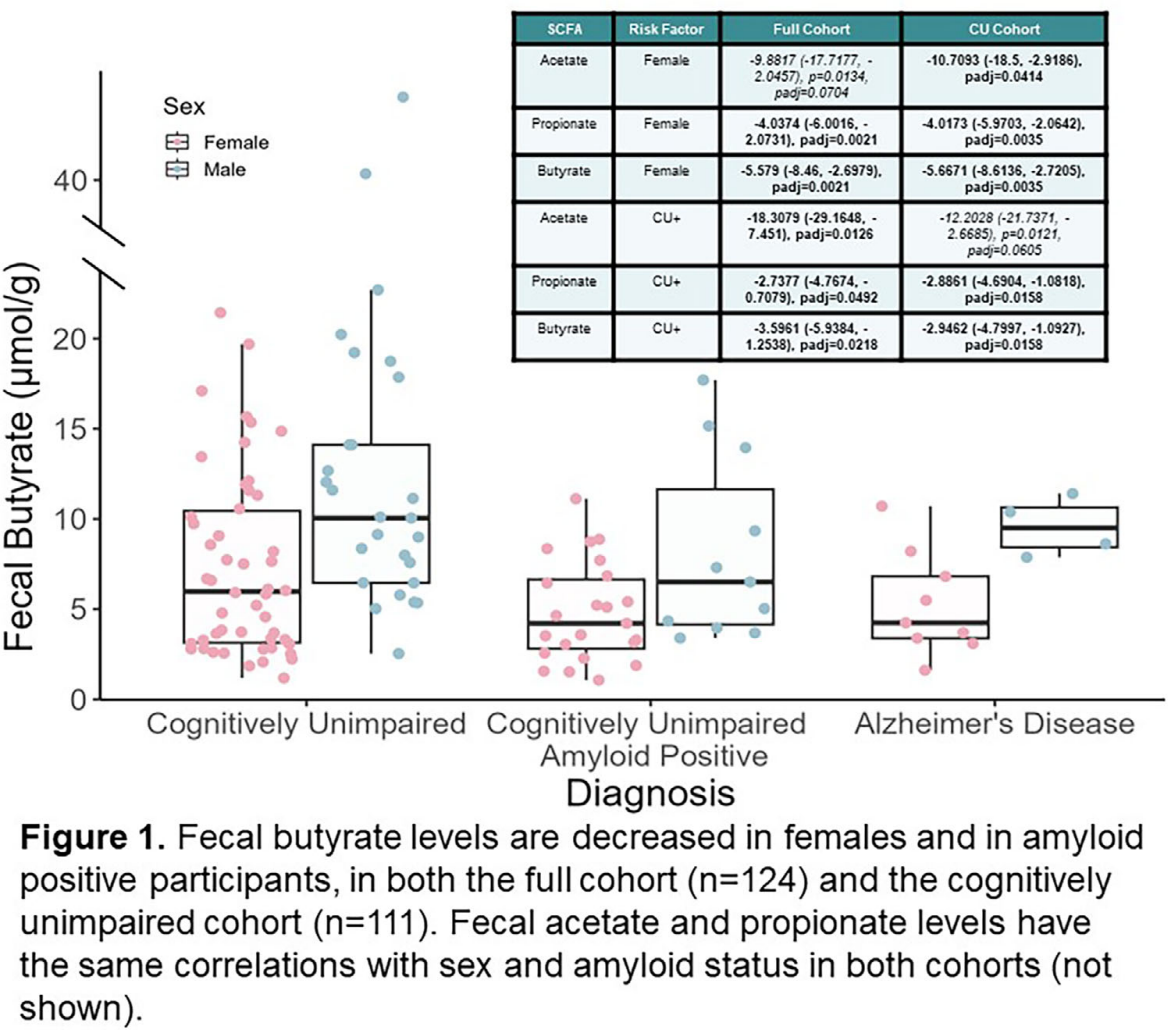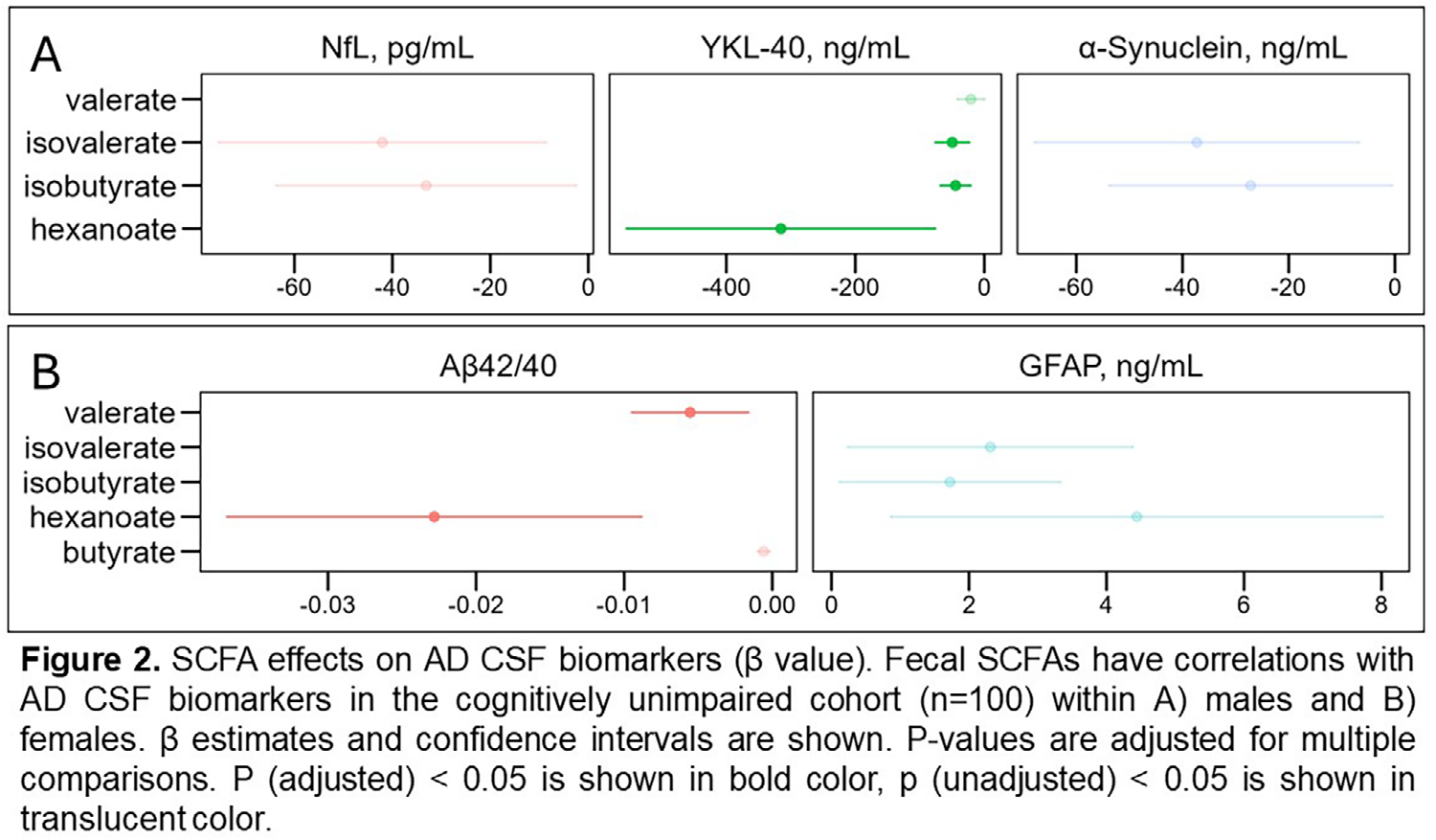# Relationships Between Microbiome‐derived Short Chain Fatty Acids and Alzheimer’s Disease Pathology Biomarkers: A Human Cohort Study

**DOI:** 10.1002/alz.094882

**Published:** 2025-01-09

**Authors:** Jessamine F Kuehn, Margo B. Heston, Matthew F. Warren, Jea Woo Kang, Sandra Harding, Nancy J Davenport‐Sis, Robert L. Kerby, Emma C. Schiffmann, Joseph L. Wheeler, Hanna M Noughani, Allison A Forst, Grace Everitt, Sushma Shankar, Alissa Mickol, Justus Zemberi, Hana Chow, Eric Zhang, Eleanor Clements, Cynthia M. Carlsson, Sterling C. Johnson, Sanjay Asthana, Henrik Zetterberg, Kaj Blennow, Barbara B. Bendlin, Federico E. Rey

**Affiliations:** ^1^ Department of Bacteriology, University of Wisconsin‐Madison, Madison, WI USA; ^2^ Wisconsin Alzheimer’s Disease Research Center, University of Wisconsin School of Medicine and Public Health, Madison, WI USA; ^3^ Department of Psychiatry and Neurochemistry, Institute of Neuroscience and Physiology, The Sahlgrenska Academy, University of Gothenburg, Mölndal, Gothenburg Sweden; ^4^ Department of Psychiatry and Neurochemistry, Institute of Neuroscience and Physiology, The Sahlgrenska Academy, University of Gothenburg, Mölndal Sweden

## Abstract

**Background:**

Alzheimer’s Disease (AD) is the most common form of dementia, and therapies that effectively halt disease progression are lacking. Short chain fatty acids (SCFAs), including acetate, propionate, and butyrate, are abundant gut bacterial metabolites produced via fermentation of dietary fibers and resistant starch. There is growing evidence that SCFAs may affect key neuropathological processes underlying AD, but their role is not well established. Understanding how gut microbial metabolites relate to AD biomarkers may help elucidate gut microbiome contributions to disease progression.

**Method:**

Here, we measured levels of several SCFAs in fecal samples collected from 124 participants in the Microbiome Alzheimer’s Research Study (MARS) using headspace gas chromatography. Participants were enrolled in the Wisconsin Alzheimer’s Disease Research Center and Wisconsin Registry for Alzheimer’s Prevention, which track preclinical disease progression in cohorts enriched for AD risk. We performed multiple regressions to identify associations between SCFA abundance and diagnosis, amyloid positivity, age, sex, BMI, APOE status, and cerebrospinal fluid (CSF) biomarkers of AD.

**Result:**

Acetate, propionate, and butyrate had significantly lower concentrations in females and in amyloid‐positive participants within both the full cohort and only cognitively unimpaired participants, suggesting that these associations may be preclinical. SCFAs had associations with several CSF biomarkers before FDR multiple comparison correction, within the full cohort and preclinically. Hexanoate and butyrate had negative associations with Aβ42/40; hexanoate had a positive association with sTREM2, a marker of microglial activation, and NfL, a marker of neurodegeneration. Isobutyrate had a negative association with YKL‐40, a marker of astrocytes. Preclinically, isobutyrate and isovalerate had a negative association with NfL, and acetate had a positive association with GFAP, a marker of CNS astrocytes. An exploratory analysis found a SCFA‐by‐sex interaction on CSF biomarkers: in males, isobutyrate, isovalerate, and hexanoate had a negative association with YKL‐40, while in females, valerate and hexanoate had a negative association with Aβ42/40. Metagenomic analysis investigated associations with SCFA production‐related genes.

**Conclusion:**

The three most abundant SCFAs (acetate, propionate, and butyrate) had lower concentrations in females and in amyloid‐positive participants in a human cohort enriched for AD risk. Fecal SCFAs have associations with AD CSF biomarkers in the same cohort.